# Understanding Medical Students’ Knowledge of Opioid Use Disorder: A Preliminary Study

**DOI:** 10.1093/geroni/igaa057.706

**Published:** 2020-12-16

**Authors:** Sweta Tewary, Ariel Kidron, Naushira Pandya, Jim Howell, Marie Florent-Caree, Annisah Ishmael, Rebecca Cherner

**Affiliations:** 1 Nova Southeastern University, Fort Lauderdale, Florida, United States; 2 NSU, Davie, Florida, United States

## Abstract

The rise of the opioid epidemic over the last two decades has increased the mortality rate, healthcare cost, and drug overdose deaths across the country. Practicing physicians are lacking in education regarding non-opioid alternatives to pain management, prevention, diagnosis, and treatment of opioid use disorder (OUD). Existing literature suggest a link between knowledge discrepancy and opioid use among clinicians resulting in patient’s abuse of opioids. Therefore, it is important to educate medical students at the start of their career. This preliminary study assesses the current knowledge and perceived skills of medical students regarding (OUD)/opioid misuse and related content in order to identify gaps and provide necessary education. The study used a pre-post survey method to understand the demographics, medical, and clinical knowledge about opioid use, abuse, and clinical knowledge regarding patient opioid overdose. The self-administered survey was administered to all students 18 years or older, M1- M4 enrolled in NSU-KPCOM. A total of 1164 students met these criteria. However, only 137 students participated in the Pre-survey collected from August 2019 to September 2019. Approximately 12% of the eligible students participated in the pre-survey. Data was analyzed using frequencies and percentages. Results of the pre-survey suggest a progressive increase in opioid knowledge from M1 to M4 years. Results of the study suggest investigating a relationship between medical education and knowledge of opioid usage, with a specific lens aimed at assessing the efficacy of opioid education during second and third years of medical school

